# Post-radiation Angiosarcoma of the Vagina: A Case Report and Literature Review

**DOI:** 10.7759/cureus.72475

**Published:** 2024-10-27

**Authors:** Joseph Sotelo, Monish Sharma, Steve Schultz

**Affiliations:** 1 Radiology, Texas College of Osteopathic Medicine, Fort Worth, USA; 2 Radiology, Radiology Associates of North Texas, Fort Worth, USA

**Keywords:** post-radiation malignancy, radiation induced angiosarcoma, rare cancers, taxol, vaginal cuff

## Abstract

Angiosarcomas are rare and aggressive mesenchymal neoplasms that can be primary or secondary to other factors such as radiation exposure. They can occur anywhere in the body but are most commonly found on the skin. Post-radiation angiosarcoma of the vagina is a very rare neoplasm, with few cases reported in the literature. We report the case of an 83-year-old female patient who developed angiosarcoma of the vagina 14 years after receiving radiation therapy for rectal carcinoma. The patient was then treated with Taxol chemotherapy and showed an excellent response to treatment, with complete remission at four years post-treatment.

## Introduction

Angiosarcomas are aggressive and rare mesenchymal neoplasms commonly presenting on the skin. Angiosarcomas are known to occur secondary to radiation; however, the majority of these cases occur in the breast, skin, and soft tissues [1]. In the past 30 years, although the incidence of angiosarcoma has risen for incompletely understood reasons, angiosarcomas of gynecologic origin remain very rare [2]. To our knowledge, there have only been 10 cases of angiosarcoma of the vagina or vulva secondary to radiation [3]. Of those cases previously reported, the prognosis has been dismal. The median survival period for angiosarcomas secondary to irradiation has been reported as 12 months [4]. We believe this may be the first documented case of post-radiation vaginal angiosarcoma with complete remission.

## Case presentation

An 83-year-old female patient with a history of robotic-assisted hysterectomy was diagnosed with rectal carcinoma in 2006. The rectal carcinoma was then removed via low anterior resection, and subsequent examination showed involvement of one lymph node. The patient then underwent radiation therapy and adjuvant chemotherapy to treat regional involvement. In 2010, four years later, the patient was found to have solitary pulmonary metastasis. This was treated with surgical resection. 

**Figure 1:**
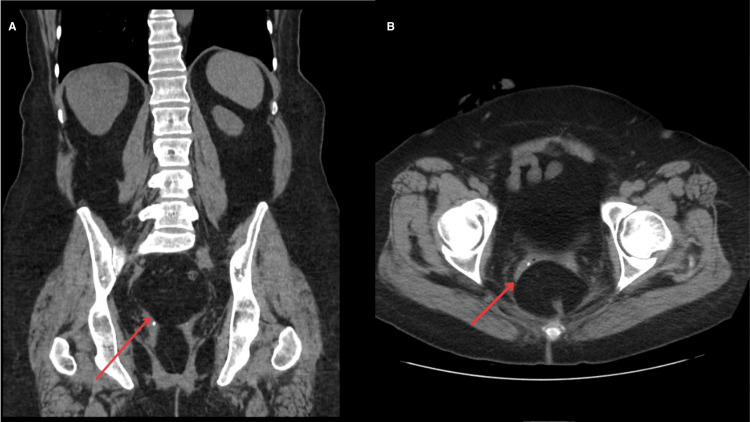
CT scans WOC pre-tumor Coronal (A) and axial (B) CT scans WOC taken in 2010 showing no gynecological involvement (red arrows). CT: computed tomography; WOC: without contrast

**Figure 2:**
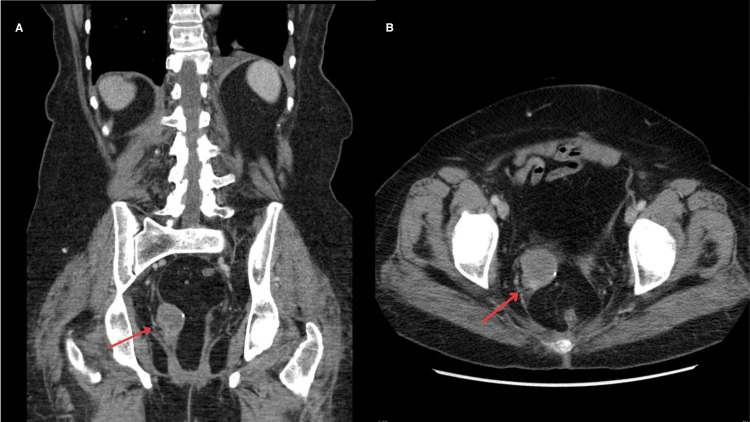
CT scans WOC with tumor at the vaginal cuff Coronal (A) and axial (B) CT scans WOC taken in 2020 showing a new tumor at the vaginal cuff (red arrows). CT: computed tomography; WOC: without contrast

After 10 years, in 2020, the patient presented for vaginal bleeding. Imaging performed in 2010, at the time of pulmonary metastasis, showed no gynecologic involvement (Figure 1A, 1B). However, a repeat computed tomography (CT) scan without contrast done in 2020 following episodes of vaginal bleeding showed a 4 cm mass at the vaginal cuff (Figure 2A, 2B). A biopsy of the vaginal mass was conducted and demonstrated high-grade angiosarcoma, believed to be secondary to radiation therapy. As the patient was not a surgical candidate, angiosarcoma was treated with Taxol chemotherapy. Despite the poor prognosis associated with angiosarcoma, the patient exhibited a significant response to treatment. Follow-up magnetic resonance imaging (MRI) performed in July 2021, September 2021, and December 2021 showed a complete response to treatment. The patient has not had a recurrence of angiosarcoma since.

## Discussion

Angiosarcomas are rare and aggressive mesenchymal neoplasms most commonly found on the skin. Although radiation therapy is a known risk factor for the development of angiosarcoma, the breast, skin, and soft tissues are the most likely sites of tumor origin. Very few cases of radiation-induced angiosarcoma of gynecologic origin have been documented. However, of the 10 reported cases of vaginal angiosarcoma, six have had a history of radiation therapy [1]. Furthermore, five-year overall survival for female genital tract angiosarcomas has been reported as 27%, making the prognosis dismal [5]. This case of an 83-year-old female patient who developed angiosarcoma 14 years after receiving radiation therapy is unique due to not only the gynecologic site of origin but also a complete response to Taxol chemotherapy, without recurrence of the tumor. 

Radiation is an established risk factor for angiosarcoma; however, an interesting finding is that eight of 12 cases of angiosarcoma of the vagina also had a previous hysterectomy [1]. In a study of 87 patients with primary cancer of the vagina, 31 had a history of prior hysterectomy. Similar to the 83-year-old patient presented in this case, the most common presenting symptom in these patients was vaginal bleeding [6]. It is important to note that squamous cell carcinoma accounts for 90% of all patients with primary vaginal cancer [7]. Traditional screening methods such as Papanicolaou smear can detect these more common cancers and precancerous lesions, indicating that post-hysterectomy vaginal screening may be beneficial. However, it is unclear how beneficial early screening would be for rare, difficult-to-diagnose cancers such as angiosarcoma, especially considering gynecologic sites of origin are uncommon. 

The primary treatment of choice for angiosarcoma is typically radical surgical excision. However, in cases such as this one, where metastasis has developed or a patient is not considered a surgical candidate, chemotherapy is considered the first-line treatment. There is no consensus in terms of which chemotherapy provides the best survival outcomes; however, taxanes, anthracyclines, and tyrosine kinase inhibitors show higher survival rates. It is thought that the varied response to chemotherapy for angiosarcoma is due to a high variability in histopathological characteristics. More aggressive tumors have more disorganized cellular architecture, a unique cellular appearance, growth rate, and differentiation, making a chemotherapeutic regimen difficult to standardize [8]. Studies conducted on the chemotherapeutic treatment of skin angiosarcoma using weekly paclitaxel increased the overall survival from eight to 19.5 months. Of the two researched cases of vaginal angiosarcoma treatment response to chemotherapy, the case with weekly paclitaxel therapy had the longest overall survival of 24 months [8]. In this case, the patient showed a complete response of angiosarcoma to Taxol therapy, indicating that Taxol therapy may lead to better survival outcomes for radiation-induced angiosarcoma of gynecologic origin. Comparatively, another report used combination chemotherapy (vincristine, dacarbazine, cyclophosphamide, and doxorubicin) and interleukin-2 to induce remission; however, the patient was disease-free for 15 months post-treatment [9].

## Conclusions

Angiosarcomas are rare mesenchymal neoplasms; however, they should always be on your differential in a previously irradiated area, for they may mimic the recurrence of the initial tumor. The mean time period for post-radiation angiosarcoma to appear is around 6-7 years, and this case shows the importance of recognizing that occurrence outside of the mean should not be overlooked. Furthermore, this case may help revolutionize and standardize the treatment for post-radiation angiosarcomas due to the complete remission found with Taxol therapy in our patient, which has been documented to have the most success in the limited literature on this topic.
